# ACES: a machine learning toolbox for clustering analysis and visualization

**DOI:** 10.1186/s12864-018-5300-y

**Published:** 2018-12-27

**Authors:** Jiangning Gao, Görel Sundström, Behrooz Torabi Moghadam, Neda Zamani, Manfred G. Grabherr

**Affiliations:** 1 Department of medical biochemistry and microbiology, Uppsala University, Uppsala, Sweden; 2 Department of Forest Genetics and Plant Physiology, Swedish University of Agricultural Sciences, Umeå, Sweden; 3 Department of immunology, genetics, and pathology, Uppsala University, Uppsala, Sweden

**Keywords:** Clustering, Data visualization, Centroid detection, Discriminative power prediction

## Abstract

**Background:**

Studies that aim at explaining phenotypes or disease susceptibility by genetic or epigenetic variants often rely on clustering methods to stratify individuals or samples. While statistical associations may point at increased risk for certain parts of the population, the ultimate goal is to make precise predictions for each individual. This necessitates tools that allow for the rapid inspection of each data point, in particular to find explanations for outliers.

**Results:**

ACES is an integrative cluster- and phenotype-browser, which implements standard clustering methods, as well as multiple visualization methods in which all sample information can be displayed quickly. In addition, ACES can automatically mine a list of phenotypes for cluster enrichment, whereby the number of clusters and their boundaries are estimated by a novel method. For visual data browsing, ACES provides a 2D or 3D PCA or Heat Map view. ACES is implemented in Java, with a focus on a user-friendly, interactive, graphical interface.

**Conclusions:**

ACES has been proven an invaluable tool for analyzing large, pre-filtered DNA methylation data sets and RNA-Sequencing data, due to its ease to link molecular markers to complex phenotypes. The source code is available from https://github.com/GrabherrGroup/ACES.

## Introduction

One fundamental challenge in modern biology and medicine is to divide samples into distinct categories, often cases and controls, based on the measurements of biomarkers in the wider sense [1]. With advances in high-throughput sequencing technologies, these markers can comprise a large number of data points, such as in whole-genome resequencing, RNA sequencing, or DNA methylation status data. Here, identifying informative sites or markers is essential, necessitating tools to quickly assess what subset of markers are associated with what phenotypes. In mathematical terms, the problem can be divided into three parts: (a) feature selection; (b) data clustering; and (c) correlating data clusters to phenotypes.

For univariate or multivariate data clustering, a number of core algorithms have been implemented and made available, such as Cluto [2], Cluster 3.0 [3] and NeAT [4]. In addition, there are numerous software packages for MATLAB and R, albeit limited to smaller datasets due to memory constraints. For cluster visualization, jClust [5] provides a graphical user interface, as does ClustVis [6], a web tool using 2D scatterplots and localizations. Mayday [7] is a powerful and distributed R-based platform for analysis and visualization, which was initially designed for microarray analyses. Likewise, The Hierarchical Clustering Explorer [8] focuses on microarrays and visualizes the data primarily as dendrograms and heat maps, similar to Clusterphile [9], which does focus on interactively exploring the data.

Unlike these tools and packages, ACES provides a full workflow guiding the user through a process that starts with a distance matrix or raw data, all the way to connecting the clustering results to a set of phenotypes. ACES is implemented using a modular design, which allows for expanding its functionality and including other tool’s algorithms in the future.

Here, we present ACES, an integrated data analysis tool that combines all the functionality outlined above. Implemented in Java, it provides an interactive graphical user interface that makes the analysis available even to non-expert users. Machine learning components aim at facilitating assaying the data, including estimating parameters, notably the number of distinct clusters [10] and other parameters specific to the algorithm, depending on the input data. ACES supports multiple input formats, providing functionality to filter and further refine the format. Given a distance or minus-log-probability matrix, ACES automatically extracts features of each identity for the following clustering analysis. As different feature extractions algorithms are applied to the original data, a distance matrix is always the resulting input for clustering. ACES implements several clustering algorithms, including hierarchical clustering [11], *k*-means [12] and DBSCAN [13], while being extendable to other methods.

For visualization, ACES reduces the dimensionality of the samples by Principle Component Analysis (PCA) [14] to plot them in a 2D or rotatable 3D view, or in Heat Maps, as shown in Fig. 3. It computes the predicitive power of each phenotype to cluster formation, and thus allows for rapidly accessing correlations between the underlying data and the sample information provided with the data.

## Implementation

**Fig. 1:**
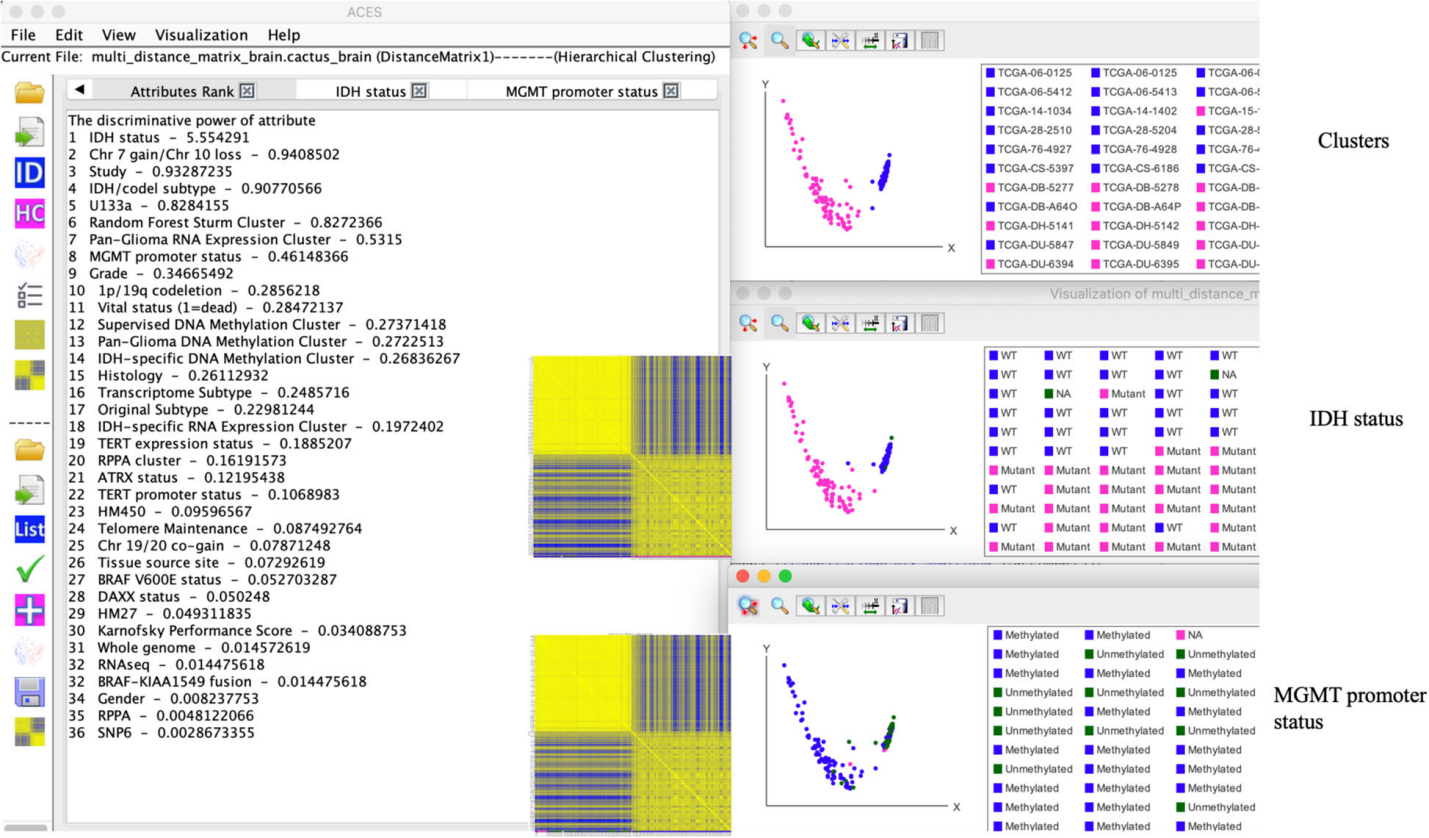
Overview of ACES. An example is used to show ACES. ACES loads a Saguaro formated file with several distance matrices, of which distance matrix 1 is chosen. Together with the clustering results shown in two colors (pink and blue), distance matrix 1 is visualized either in a 2D scatter plot or Heat Map view. The heat map is reordered by the clustering results. Also, ACES can load the corresponding attributes and predict their discriminative power shown on the screen, using the clusters. According to the prediction, IDH status and MGMT promoter status are selected and then visualized in the bottom scatter plots, as well as at the bottom line of localization in the heat map. The points are colored by their selected attribute label and clickable to view a full set of attribute information, which clearly demonstrate the relationship between selected attributes and the clusters results

ACES is platform independent, implemented in Java, and sets emphasis on user-friendliness for scientists in biology and medicine, both experts and non-experts in statistical data processing. A schematic view of the workflow is shown in Fig. 1: ACES loads either raw data, or one or more pre-computed distance or probability matrices. ACES is file-format compatible with Saguaro [15], yet supports multiple matrix formats, including phylip [16] (for more details, see the documentation at https://grabherrgroup.github.io/ACES/). If raw data is given, ACES computes a distance matrix based on the Manhattan Distance, Euclidean Distance, or Pearson correlation coefficient. ACES next applies clustering and estimates the number of clusters, as well as their centroids. If attributes (phenotypes, disease categories, medical data, etc.) are available, ACES predicts and ranks these attributes against the clusters. Attributes can then be selected and displayed either in the 2D/3D scatter plot or Heat Map view. Each data point is clickable and displays a full set of attribute information.

### Clustering

ACES implements both agglomerative hierarchical and *k*-means clustering. For hierarchical clustering, each sample starts in its own cluster, and pairs of clusters are merged as one moves up the hierarchy, which builds clusters incrementally. While *k*-means clustering aims to partition the samples into *k* clusters, which uses cluster centers to model the samples so that each sample belongs to the cluster with the nearest mean. As all the samples will be grouped into certain clusters, the number of clusters is predefined. In comparison, using hierarchical clustering, the sample data is first computed into a tree topology before the number of clusters is determined. For ease of use, ACES automatically estimates the number of clusters and the initial centroids by a novel cluster centroid localization algorithm implemented in ACES (see “Cluster centroid detection” section).

In addition, ACES implements DBSCAN, which is a density-based clustering algorithm that groups identities that are closely packed, while detecting and marking outliers. DBSCAN requires two parameters: the radius (*eps*), and the minimum number of identities (*minPts*) required to form a dense region. A sample is defined as a core sample if at least *minPts* points are within distance *eps* of it. All other samples within this radius are directly reachable from this sample. For any two core samples, the reachability can be generated by the common core samples. All the samples that are not reachable from any other samples are considered as outliers. Therefore, the parameters *eps* and *minPts* can be decreased to make more clusters and increased for less clusters.

While DBSCAN does not require the specific number of clusters a priori, *eps* and *minPts* need to be determined beforehand. ACES provides automatic estimates by default, computed from the distance matrix, where the default *eps* requires that at most 25% distance values of all the identities pairs are lower than *eps*. *minPts* is set by the number of identities.

### Cluster centroid detection

**Fig. 2:**
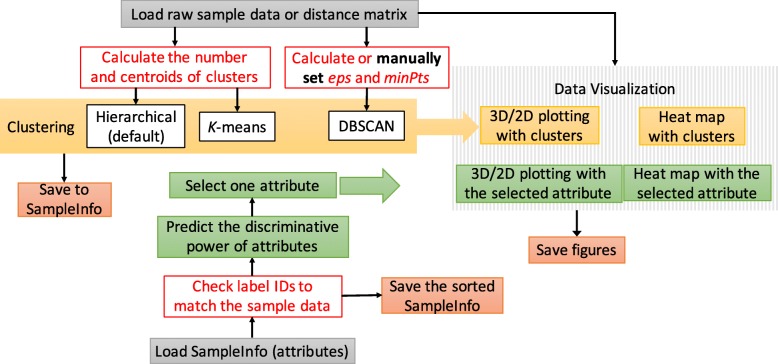
The workflow of ACES. ACES first reads the raw sample data file or distance matrix, and then automatically calculates the number and the potential centroids of clusters. Hierarchical clustering is set as the default, also allowing for *k*-means and DBSCAN. Initial input parameters are automatically estimated. To demonstrate the relationships among the samples together with the clustering results, the samples are downsized by PCA and visualized in 2D or 3D plots, colored by their cluster labels. Alternatively, the distance matrix is reordered for heat map visualization to show the clusters. ACES provides functionality to analyze data samples with multiple phenotypes to best explain the clustering: ACES automatically extracts and sorts all phenotypes/attributes and ranks them by consistency with the biomarker data, i.e. the discriminative power of each attribute is matched to the clusters in the data. The matches are then visualized in 2D/3D PCA plots as well as at the bottom of heat map, colored by attribute labels

The similarity of two identities is represented by the distance. To localize all candidate centroid identities, the most salient identity (*SI*) is first found. *SI* is defined as the identity that contains the most diverse similarities to other identities. For this kind of identity, the variance of the similarities range must be large enough to ensure that there is a clear boundary between itself and the other potential centroid identities. To this end, for each identity, all distances to the target identity are used to calculate the distance vector and the standard deviation (*Std*_*i*_). The identity with the highest *Std*_*max*_ is selected as the *SI*, which is considered as the first candidate centroid. As shown in Fig. 2, all identities are represented as black points in the 3D plot. The red point is finally selected as the *SI*.

Given the *SI* found in the initial step, the remaining centroids (shown as green and blue in Fig. 2) are localized by the searching window defined for each identity. For the *i*^*th*^ identity, a radius *R*_*i*_ is set to build a circle searching window that contains all neighboring identities on the basis of the distance matrix. Given that *DM*_*ij*_ is the distance between the *i*^*th*^ identity and *j*^*th*^ identity, one of the distances (*DM*_*i*1_, *DM*_*i*2_, ⋯, *DM*_*in*_), which is higher than most distances is set to *R*_*i*_ to ensure that at least 90% identities are within the *i*^*th*^ searching window. Identities that are not within the searching window of *i*^*th*^ identity are defined as the outliers of *i*^*th*^ identity. Also, all centroid identities should possess higher *Std*_*i*_ as defined above to ensure the variance of their similarities from other identities is large. Therefore, the outliers of *SI* with high *Std* are set as initial potential centroids.

For each new potential centroid, if all the detected centroids are its outliers, it is considered a new centroid, which means the centroid should be the outlier of the other centroids. As described in Fig. 2: the searching window of *SI*, shown as red circle is first applied, and the farthest identity (the green point in Fig. 2) with high *Std* is selected as the second centroid. Then, all common outliers of these two detected centroids with high *Std* are used for comparison, and the blue point is found as the third centroid. These detected centroids are then used as the initial parameters of *k*-means clustering, while the number of centroids are used for both hierarchical and *k*-means clustering.

### Correlating clusters and attributes

ACES first determines the number of distinct and discrete labels in each attribute, and for each attribute containing no more than *N*_*a*_ unique labels, it sets a (*N*_*a*_-1) dimenstional feature vector for each unique labels to ensure the distance between each two unique labels pair is the same. ACES then computes a statistically weighted score (*S*) for each attribute by

$$\begin{array}{*{20}l} S_{b}&=\sum\limits_{i=1}^{k} N_{i}(\mu_{i}-\mu) (\mu_{i}-\mu)^{T}\\ S_{w} &= \sum\limits_{i=1}^{k} S_{wi} = \sum\limits_{i=1}^{k} \sum\limits_{x\in X_{i}} (x-\mu_{i})(x-\mu_{i})^{T}\\ S &= \frac{S_{b}}{S_{w}} \end{array} $$

where *k* is the number of clusters, and *X*_*i*_ represents all the samples within *i*^*th*^ cluster. *N*_*i*_ and *μ*_*i*_ are the number and mean value of samples in *i*^*th*^ cluster. *μ* is the mean value of all the samples. *S*_*b*_ and *S*_*w*_ are the standard deviation between clusters and within clusters, respectively.

### 2D/3D Scatter and heap map view

**Fig. 3:**
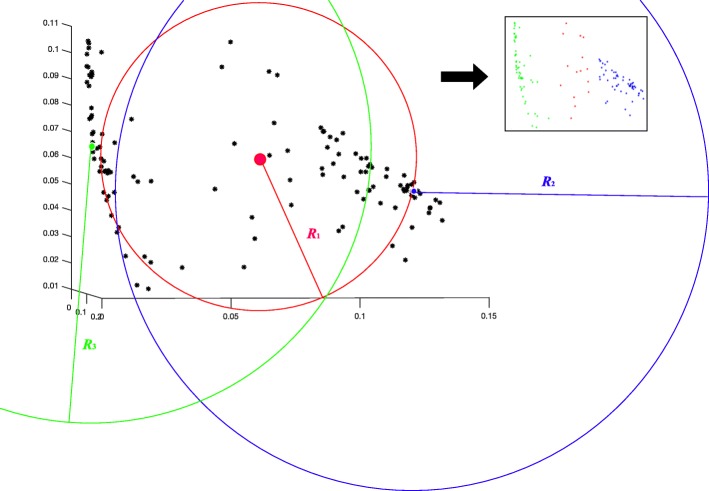
Cluster centroid detection. Using the distance matrix or raw data, all the identities are visualized as black points in the 3D PCA scatter plot on the left. The standard deviation of each identity is calculated by its corresponding row or column in the distance matrix. The red point with the highest standard deviation is first detected as the *SI*. Using the red searching window of *SI*, the green point is localized as the second centroid. As the common outliers of both red and green circles, the blue point is found as the third centroid. Using these detected centroids, the clustering results are shown on the right

In 2D or rotatable 3D view, all samples are either color coded by their repective clusters, or a selected attribute (Fig. 3). Multiple views can be shown simultaneously, and each data point displays complete attribute information when clicked. All images can be exported in Scalable Vector Graphics in production quality. ACES shows both the Heat Map values, as well as the attributes on the left column and bottom row (Fig. 3).

## Results

### DNA methylation

DNA methylation (DNAm) is an epigenetic mechanism that can control gene expression. It has been shown that DNAm modification of certain sites are directly linked to cancer [17]. We extracted data from 65 and 100 samples with glioblastoma multiforme and lower grade glioma respectively from the Cancer Gemone Atlas [18, 19], and applied the unsupervised method Saguaro [15] to segment the genome into distinct regions, yielding seven distance matrices exhibiting different classifications. To interpret the results, we applied the following methods.

### PCA 2D/3D visualization

For each distance matrix, we obtained main components using PCA and plotted the samples/distances in a 2D/3D view. For one of the matrices, ACES finds two clusters, and predicts the highest concordance with IDH mutation status, followed by histological assessment (Lower Grade Glioma or Glioblastoma multiforme), in accordance with the original study [20].

### Functional heat map

ACES provides two interfaces to generate a Heat Map: (a) the distance matrix before clustering and with the label IDs; (b) a distance matrix resorted accoding to the clustering results. In addition, ACES can also merge the attribute into the Heat Map by adding an extra row on the bottom, marking colors consistent with the PCA attributes visualization.

### Clustering results

**Fig. 4:**
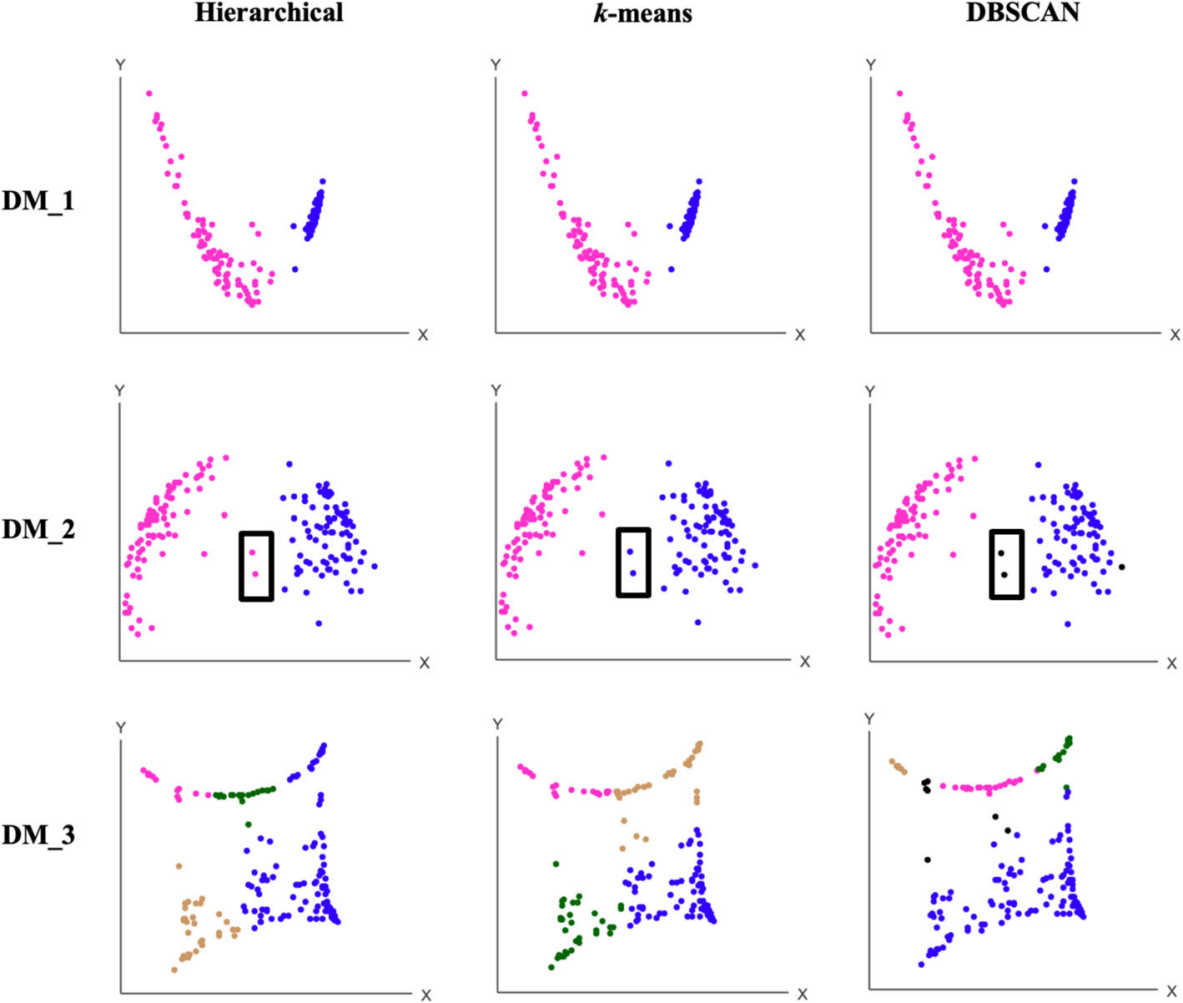
Clustering results shown by the PCA 2D scatter. Three distance matrices generated from brain DNA methylation data are shown by three methods. From the left to right: Hierarchical, *k*-means, and DBSCAN

We used three distance matrices from the brain cancer data to demonstrate the three clustering methods implemented in ACES. The PCA 2D visualization represents the samples as two-dimensional points, colored by the clustering or labels (Fig. 4). For each distance matrix, clusters found by hierarchical, *k*-means and DBSCAN algorithms are shown in each row. The points in black are considered as outliers by DBSCAN, indicating that these points could not be reliably assgined to any cluster.

As shown in Fig. 4, ACES groups the original samples without any predefined parameters. The parameters are automatically estimated by the data samples or respective distance matrix. Further, for data samples clustering within clear boundaries, all clustering algorithms perform well, exemplified by Distance Matrices 1 and 2 in Fig. 4. Specifically, the three clustering algorithms produce the same results for Distance Matrix 1, shown on the top. In Distance Matrix 2, all samples are categorized as the same groups, except for two points close to the boundary in the middle. Hierarchical and *k*-means categorize them into different groups, while DBSCAN considers them as outliers, according to the parameters that are automatically computed by ACES. For Distance Matrix 3, shown in the bottom row, the three clustering algorithms generate different results, as there are no clear boundaries among groups.

**Fig. 5:**
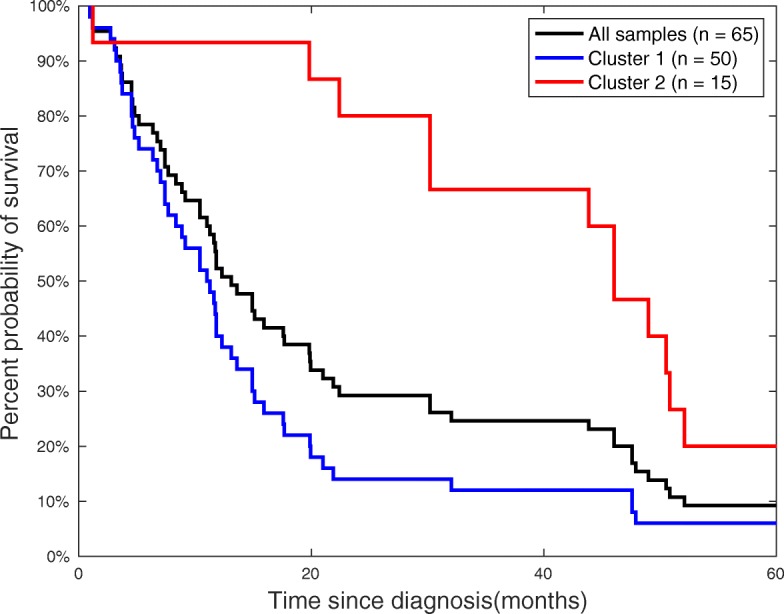
Survival Analysis Using Distance Matrix 1. Shown are the survival rates of 65 people who died within 60 months (black), compared to the hierarchical clusters, where cluster 2 indicates longer survival compared to the patients in cluster 1

Figure 5 shows a Kaplan-Meier survival plot for the clusters in Distance Matrix 1, which is consistent with the findings of the original study [20] in that IDH mutation status constitutes a better predictor for survival than histology. Specifically, there are 65 people who died within 60 months in this survival analysis. Using hierarchical clustering, patients in cluster 2 (all IDH mutants) exhibit longer survival compared to those in cluster 1 (all IDH wildtype).

### RNA-Seq expression in pancreatic islets from Type-I diabetes patients and controls

The dataset published by Krogvold et al. [21] consists of RNA sequencing from pancreatic tissue from six type 1 diabetic patients (samples DIVID1 through DIVID6), two brain dead organ donors that died at the onset of type 1 diabetes (samples H911 and H1204), and three brain dead non-diabetic organ donors (samples H1778, H1499, and H1530). Here, we choose to use the RPKM values as the raw data input, using the same gene pathways as in the original study, which are the “complement system” and the “insulin secretion pathway”.

**Fig. 6:**
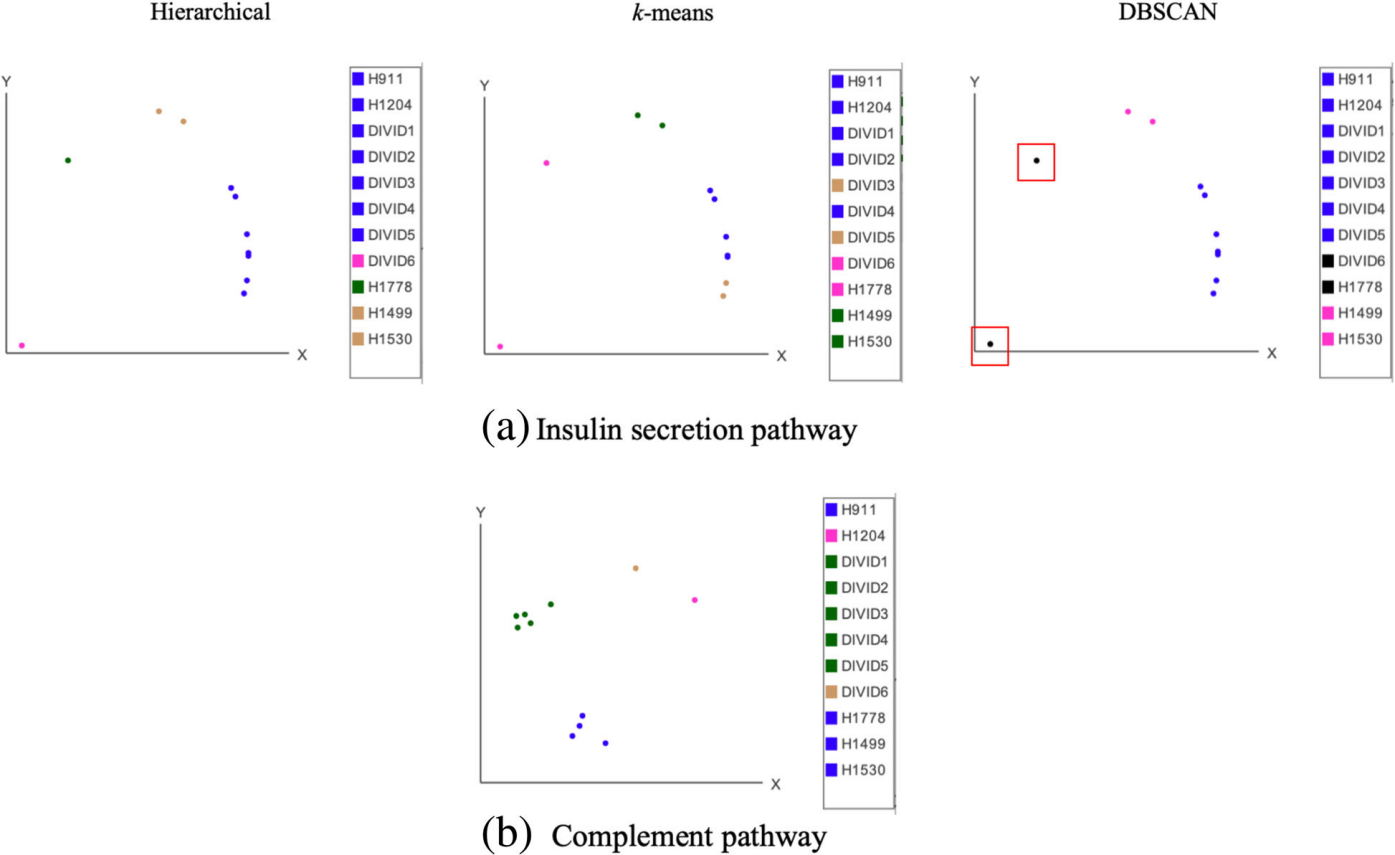
Type-I diabetes patients and controls shown by PCA 2D scatter. Shown are three clustering methods for the live diabetic samples (DIVID1 through DIVID6), brain dead early-onset diabetics (H911 and H1204), and three brain dead controls (H1778, H1499, and H1530), for genes in the insulin secretion pathway, which should separate by cases and controls (**a**). By contrast, the complement pathway, which is unrelated to insulin metabolism, separates samples by brain dead versus live patients (**b**). Sample DIVID6 was identified as outlier in the original study

On the insulin secretion pathway (Fig. 6a left), hierarchical clustering finds one cluster that contains all diabetic samples, except for DIVID6, which was also an outlier in the original study [22]. While *k*-means (Fig. 6a middle) groups the samples in two clusters, these two samples are classified as outliers in the DBSCAN clustering results based on the parameters that were automatically calculated by ACES (Fig. 6a right). While the granularity of ACES results in more clusters with each method, the sample grouping produced by ACES based on hierarchical clustering is identical to the orignial study, which also applied hierarchical clustering. Figure 6b, which shows the complement pathway, demonstrates similar differences while comparing the clustering methods. However, the results show that the samples have been clustered by alive and brain dead patients and regardless of their diabetic status, identical to the results in the original study.

## Conclusion

Analyzing medical or biological data benefits from quick and interactive tools to quickly assay the data. Here, we present ACES, a visual browser specifically geared towards comparing phenotypes or medical diagnoses to the underlying genetic, epigenetic, or proteomic data. ACES implements a number of features that makes it suitable even by non-expert users, by encapsulating clustering algorithms beneath a layer that estimates critical parameters, and by automatically linking cluster results to different kinds of sample meta-information. In addition, being implemented in Java rather than R or matlab, ACES is directly accessible to users not familiar with those envorinments. We expect that ACES will contribute significantly to biomedical research in many areas and diseases.

## Availability and requirements

**Project name:** ACES

**Project home page:** https://github.com/GrabherrGroup/ACES

**Operating system(s):** Platform independent

**Programming language:** Java

**Other requirements:** Tested under Java SE 1.8.0

**License:** GNU GPL Version 3

**Any restrictions to use by non-academics:** no

## Abbreviations

DNAm: DNA methylation
PCA: Principle component analysis
SI: Salient identity
